# Collectanea

**Published:** 1831-07

**Authors:** 


### COLLECTANEA.

**Floriferis ut apes in saltibus omnia libant,**
**Omnia nos, itidem, depascimur aurea dicta.**

### PATHOLOGY.

*Influence of Atmospheric Electricity on the Eyes.* We see peculiar appearances in weak and morbidly sensitive eyes, before the breaking out of a violent storm, shewing the positive influence of an atmosphere which has now attained its maximum of electricity; and I am acquainted with several persons who are able, from a certain premonitory feeling of their weak eyes, to predict a thunder-storm infallibly, a considerable time beforehand. As every experienced oculist is convinced of the bad effects of an atmosphere of this kind, he will never extract the cataract during the approach of a thunder-storm.—Beer, *Lehre von den Augenkrankheiten*, § 72.

---

*Poisoning by the Sebacic Acid of Goose-Grease.* On the 2d of April, 1829, Dr. Siedler was called to attend MM. H—, and their children. On his arrival he found the two brothers H—, one aged thirty-one, the second twenty-eight years, and the two children of the first, one a girl, æt. four, the other a boy, æt. two and a half, all presenting the following symptoms: Cold sweat, anxiety, vertigo, general paleness and prostration of strength, eyes sunken and pupils dilated; burning pain was felt in the lower part of the belly, increased by pressure; violent vomiting, succeeded by ardent thirst, for which the patients had drunk large quantities of milk, which was thrown up without producing any effect; tongue dry; involuntary discharge of urine and fæces.

The eldest brother was insensible for six minutes, his respiration was scarcely visible, his pulse imperceptible, and the heart's action exceedingly weak. The second brother had vomited blood several times, but he experienced less abdominal pain than the others. In the little boy the globes of the eyes were turned upwards, the lips livid, and the pulse scarcely sensible. Lastly, the symptoms in the little girl were the mildest of all. M. Siedler suspected at once that these accidents were occasioned by the use of a certain quantity of goose-grease which had been employed in the preparation of some meat, of which the four patients had eaten shortly before the symptoms began. An emulsion containing hyoscyamus was prescribed, and on the 9th of April all had recovered.

The vomited matters were subjected to chemical analysis: they were strongly acid, but contained no metallic poison; but the following facts induced Dr. Siedler to attribute the illness to the effects of sebacic acid. The lady of the house had made use of goose-grease to dress some veal, and all the persons who partook of the dish fell quickly sick; the lady herself, who had barely tasted it, felt it so disagreeable that she took no more. None of the grease which was suspected to have caused the accident remained for examination, the pot which contained it having been entirely emptied and cleaned out, but on examining the same kind of grease contained in three other pots, it was found to exhale a strong repulsive odour, and it reddened strongly blue paper tinged by turnsole. Three ounces of this grease were given to a vigorous well-formed dog; an hour after, his extremities became violently convulsed, he cried piteously, he refused to eat, his eyes were suffused, pupils dilated, skin cold, and arterial pulsations scarcely perceptible. In this state he continued for thirty hours, after which he slowly recovered.—*Lancet*, from *Hufeland's Journal.*

---

*The Dependence of Hysteria upon Cerebral and Spinal Irritation.* By John Darwall, m.d., Physician to the Birmingham Dispensary. (*Midland Med. Rep.*)

That many of those anomalous symptoms which have been comprehended under the title of hysteria, depend essentially upon some affection of the brain and spinal medulla, seems now to be generally acknowledged. It is not, however, very frequently, that such evidence can be obtained of the correctness of an opinion, as the two following cases afford. In both of them there was manifest affection of the spine: in both there were the symptoms which are commonly known as nervous; and in both the latter disappeared as the former affection diminished. I shall give the cases as they appear in my case-book.

January 10, 1831. Master H. O. æt. fourteen. Dizziness, pain across the forehead, noise in the ear coming on suddenly, preventing his hearing. Dyspnœa; palpitation of the heart; no particular sound with the stethoscope; much tenderness over the region of the heart, and over the left side; spirits very variable, at times exceedingly low; pulse small.

The spine is much curved to the right side, so that the space between the vertebral column and the base of the scapula is much greater on the left, than it is on the right. Great tenderness over the seventh and eighth dorsal vertebræ. Bowels costive; urine natural; appetite good.

Has been unwell for twelve months, and always felt very giddy at night. Imponantur Hirudines duodecim vertebris dolentibus. ℞. Hydrarg. Submuriatis, gr. ii.; Pulv. Rhæi, Pulv. Jalapæ, *aa* gr. iij. fiat pulvis 4ta quâque horâ sumendus, donec alvus solvatur.

To assume the reclining posture whenever in the house, and not to suspend it for more than half an hour at a time, nor more than two hours during the whole day. To walk out daily.

13th. Has had less palpitation of the heart. The breath has been very bad. There is still tenderness in the spine, and in a spot between the heart and the clavicle, pressure upon which excites the action of the heart, although the respiration is very strong beneath. No cough. The leeches bled well, and the medicine brought away much offensive dark-coloured fæces. ℞. Infusi Calumbæ; Infusi Gentianæ; Infusi Sennæ, *aa* ℥ss. ter die. ℞. Pil. Hydrarg. gr. v. Fiat pil. 2ndâ quâque nocte sumenda.

February 3. Dyspnœa much the same. Has had scarcely any palpitation nor dizziness since the last report. No tenderness over the heart. Bowels open; appetite good; spirits more equable. The spine is not so distorted, but the eleventh dorsal vertebræ is much more depressed than the rest, and is tender, as also is the twelfth. Admoveatur vertebris dorsi emplastrum lyttæ. Cont. med.

March 10. In every respect better. The curvature of the spine has almost disappeared. Cont. medicamenta.

April 8. Says he is quite well; that he has no palpitation, except after great exercise; scarcely any curvature of the spine remains.

February 15, 1831. Ann G. æt. twenty-one, serves in a shop. Headach, giddiness, tinnitus aurium, low spirited, palpitation of the heart, dyspnœa, not commonly any cough. Flatulence, swelled after eating. Bowels very costive; sometimes three and four days elapse between each evacuation. Catamenia regular as to time, but last two days only; very black and painful. Slight leucorrhœa. Urine hysterical; at times very scanty and turbid, at others very copious and limpid. Pain over the region of the heart, with considerable tenderness on this spot. The spine is curved laterally, so that the middle dorsal vertebræ are within half an inch of the right scapula. Tongue reddish and dry in the morning. Pulse small and rapid. No tenderness of the spine. The spine has been curved four or five years, and the other symptoms have been present the whole time; but she has been much worse within the last month. Respiration with the stethoscope perfectly natural.

To lie down, and never to leave the recumbent posture for more than half an hour at a time; to walk out daily; regular plain diet. ℞. Pilulæ Aloes comp., Pilulæ Hydrarg. S. C. *aa* ʒss. Divide massam in pilulas xii., sumat iij. unâ quâque nocte. ℞. Infusi Calumbæ ℥viij.: Spiritus Ammoniæ Arom. ʒij. M. sumat coch. iij. larga ter die.

22d. Has lain down almost constantly, as directed, and thinks herself rather better; but the symptoms are still present, though, perhaps, in a less degree. Bowels open twice a day; evacuations very offensive; catamenia are come on this morning, but she has at present no pain, and they are less black. Cont. medicamenta.

March 1. Better generally; catamenia were less painful. Has had much less pain at the heart, and there is less tenderness; tongue cleaner; less leucorrhœa; pulse eighty-four; much less palpitation. Cont. medicamenta atque regimen victus.

8th. Palpitation of the heart relieved; breath better; less swelled after taking food; bowels open; evacuations dark-coloured and offensive; rather relaxed. Upon the whole is better. The tenderness over the region of the heart is quite gone.

℞. Decoct. Aloes comp; Infusi Anthemidis; Infusi Cascarillæ, *aa* ℥ss. ter die. ℞. Pilulæ Hydrar. gr. v. fiat pilula 2ndâ quâque nocte sumenda.

15th. In every respect better; much less palpitation; less headach; less dyspnœa; no leucorrhœa; not swelled after taking food; evacuations rather costive, still dark-coloured, and offensive. Considerable flatulence; tongue more natural; appetite better; pulse eighty-four, small, not so easily hurried; the curvature of the spine is less. Cont. medicamenta, &c.

22d. The catamenia have been present, but only lasted one day, and were very scanty. The bowels are open only once in two days, the appetite at the same time being good. There is this morning considerable palpitation of the heart, with irregular pulse. Spirits very low. Much tenderness over the seventh dorsal vertebra. The catamenia were very black and painful. Admoveantur hirudines duodecim vertebræ dolentis; adde Misturæ Magnes. Sul. ℥i.

25th. The leeches bled well: has had very little palpitation of the heart since; spirits better; bowels open twice or three times a day ; pulse ninety-two, regular; no tenderness now in the spine; appetite good. Cont. medicamenta.

29th. The breathing is still difficult, but only when moving. Says that she is much better: has neither giddiness, headach, nor noise in the ears; palpitation of the heart only when she walks; bowels open when she takes the pills, not otherwise. Cont. medicamenta.

This patient is still under treatment, and the spine, though very much curved, is far less so than when she first applied for assistance. Indeed, considering the time which has elapsed from the commencement of the distortion of the vertebral column, it cannot be reasonably expected that it will ever entirely recover the perpendicular direction, as the bodies of the vertebræ have probably in some degree fitted themselves, by this time, to their present situation. It is, however, particularly worthy of remark, that while, in the first case, every symptom has disappeared with the return of the spine to its natural state, and that the tenderness and palpitation were lessened by leeches to the two painful vertebræ; in the latter, one of the vertebræ became tender at the very time that there was an aggravation of the general symptoms, which were also relieved by local depletion.

The next case I shall relate, is more completely a case of hysteria, and was produced by repeated hæmorrhage; nor is the immediate connexion of the symptoms with the brain and spinal chord so evident: still the relief afforded by the treatment compels us to refer it to this origin.

March 1, 1830. Mrs. E. æt. thirty-five, has been married seven years; has had two living children; has miscarried four times within the last six months; miscarried the last time on the 6th of November. Pain in the cervical vertebræ; pressure on the fifth and sixth produces dyspnœa. Pain in the side, increased by pressure; is very hysterical; weight at the stomach after eating; frequent menorrhagia; catamenia now present. Towards night, she always becomes very ill; feels best in the morning. Has been subject to pain in the side for many years; twelve months ago, had contractions of the hands, with frequent twitchings of the limbs. Suffers much from leucorrhœa and prurigo vaginæ. Admoveatur emplastrum lyttæ nuchæ. ℞. Sulphatis Quininæ, ʒss.; Mist. Camphoræ, ℥viii. M. sumat coch. iij. larga ter die. ℞. Pilulæ Aloes comp. ʒi. in pilulas xij. sumat iij. pro re natâ. Lavationes frigidæ pudendis admoveantur.

4th. Feels better; pain in the side less continuous; less dyspnœa. The blister has been very painful, and has risen well. Less weight at the stomach; bowels have been open without the pills. Catamenia less profuse than usual, and now disappearing; pulse eighty-one, more equable.

11th. In every respect better. The pain in the cervical vertebræ is gone, as is also the pain in the side: complains of weakness at the stomach. Her bowels were rather costive, and on the 9th she took two pills, which operated severely yesterday, and last night she was rather low. Catamenia have disappeared, and she has much less leucorrhœa. Pulse eighty-four, weak. Cont. medicamenta.

18th. Convalescent. I have seen her frequently since, and she has had no relapse.

It is right to remark that, in the treatment of this patient, much stress was laid upon her diet and regimen. All diluents were forbidden, and her diet was directed to be plain and good, but not rich. There is certainly no mistake greater in many of these cases, than placing patients upon a low diet: they, under such a system, become more nervous, the stomach becomes less and less able to digest solid food, the whole system is weakened, and, should an inflammatory disease attack them under such circumstances, they instantly succumb.

Exercise, also, was strongly enjoined, but with care that it was not carried sufficiently far to induce exhaustion. It requires much caution in this respect, for, if exhaustion ensue, all the symptoms are aggravated. It is also useful to direct patients suffering from this affection, frequently to lie down during the day, to rise early in the morning, and, as they experience fatigue, to recline for about half an hour at a time. In this manner they are enabled, by degrees, to take more exercise without weariness, till they find it unnecesary to lie down at all during the day. I am indebted for the following very important case to my friend Mr. Russell, of this town: it requires no comment.

I first saw Miss G. Sept. 29, 1830. She is a delicate woman, thirty-five. She was in bed, and was complaining of a severe pain in her side, which affected her breathing so much, that she could only draw half inspirations. Her pulse was quick, but there was no fulness about it; her tongue moist, and covered with a whitish fur; she had no cough. Her great suffering arose from not having the power to draw in a natural inspiration; hence her breathing was short and laborious. She was regular; her bowels costive. She had had similar attacks before, for which she had been bled. I considered the attack of a spasmodic nature, affecting the external muscles of respiration. She took purgative and antimonial medicines, and warm fomentations were applied to the head. The attack gradually subsided, and I discontinued attending her on October 11th.

February 27th. I was again called to this young woman. I found her labouring under exactly the same symptoms described above. Similar remedies were prescribed, with the addition of eight leeches to the scrob.-cordis. These remedies gave little or no relief, and the spasms, with a quick irritable pulse, continued: it occurred to me that it was probably a case of spinal irritation, and, on examination, I found she complained of great tenderness on pressing the upper dorsal vertebræ. I directed ten leeches to be applied to the spine: they were applied, and they bled very freely. She found almost immediate relief, and has continued free from pain ever since.

The dependence of mastodynia, occasionally, upon an affection of the nerves at their root, has not been so much noticed as might have been expected after the appearance of Mr. Teale's book: the following cases are additional proofs of the correctness of his opinion.

January 25th, 1830. Mrs. Mascall, æt. twenty-five: she had suffered very severely for a year with pain in the breast, but there was neither lump nor hardness. There was much tenderness in the superior dorsal vertebræ. I first saw her on the 14th of this month, and ordered her an aperient. On the 17th, she returned again: the medicine had operated well, but she experienced no relief in the breast. She was ordered to be cupped over the painful vertebræ, which was done the same day: the pain was relieved during the cupping, and she has scarcely had any pain since. She has general dyspeptic symptoms, which are also much relieved.

February. Has had no return of pain in the breast: has not been so free from pain for twelve months. Convalescent. She had no return after this.

April 1, 1830. Mrs. Norton. She had had pain in the breast for four months, and there was distinct tenderness in the fourth dorsal vertebra. A blister was applied to the spine, and the pain ceased immediately after the rising of the blister, and four days after she was quite free from pain. I saw her some weeks after this time, and she had had no return.

I have another case under my care at the present time: she is a servant girl, and has suffered from severe mastodynia for several months. There was considerable tenderness in the fifth and sixth dorsal vertebra, to which part eighteen leeches were applied. The pain in the breast ceased for several days, but has slightly returned within the last week. She is still under treatment.

### CHEMISTRY.

*Test for distinguishing different kinds of Rhubarb.* According to M. Geiger, the ioduretted hydriodic acid produces different colours in different kinds of rhubarb, which enables us to distinguish them. Thus it gives to Muscovy rhubarb a green tinge, to China rhubarb a brown tinge, to English rhubarb a deep red colour, and to that of France a blue one.—*Journal de Chimie Médicale.*

### NATURAL HISTORY.

*Mexican Domestic Bees,* (*Melipona Beechei.*) Captain Beechey, when at Xalisco, obtained two hives constructed by these bees, which he brought to England in H. M. S. Blossom. One of them has been presented to M. Huber, and the other to the Linnean Society. They are formed of hollow trees, a portion of which, of between two and three feet in length, has been cut off, and a hole is bored through the sides into the hollows at about the middle, and the ends of the hives stopped up with clay. These hives are usually suspended on a tree in a horizontal position, with the opening into the cavity directed also horizontally, and are speedily taken possession of by the bees. Their interior arrangement differs materially from that of the European bee, some of the layers of the comb assuming a vertical and some a horizontal position, the cells of the latter being most numerous. All the combs, both vertical and horizontal, are composed of a single series of cells applied laterally to each other, and not, as in the European bee-hive, of two series, the one applied against the extremities of the other. The cells appear destined solely for the habitation of the young bees. The combs are placed together, at some distance from the opening of the hives, and surrounding them are several layers of wax, as thin as paper, irregular in their form, and placed at some little distance from each other: externally are placed the sacs for containing the honey, which are generally large and rounded in form. They vary in size, some of them exceeding an inch and a half in diameter. They are supported by processes of wax from the wood of the cavity, or from each other, and are frequently placed side by side; but their disposition is altogether irregular, and bears some resemblance to that of a bunch of grapes. Some of the honey sacs are placed apart from the others, and form a distinct cluster.

From this irregular position of the honey sacs, a most important advantage is gained by the cultivators of the Mexican hive bee, as, in order to possess themselves of the honey, all that is necessary is, to remove the plug from the end of the cavity employed as a hive, and to introduce the hand and withdraw the honey. The store of the laborious bee is thus transferred to the proprietor of the hive, without injuring, and almost without disturbing, its inhabitants. The end of the hive is then again stopped up, and the bees hasten to lay in a fresh store of honey. A hive treated in this way affords, during the summer, at least two harvests.

The bee itself, by which this nest is constructed, is smaller than the European hive bee; its abdomen especially being much shorter. It is distinguished also from the European race of hive bees by the form of the first joint of its hinder tarsi, which is that of a triangle, with its apex applied to the tibia. Its technical characters are intermediate between the two genera Melipona and Trigona of M. Latreille, one of the mandibles being toothed, and the other nearly entire. It has a leaning towards the Trigona, but its general appearance is entirely that of a Melipona, approaching very closely to that of *Melipona favosa*, Latr., *Apis favosa*, Fab.

Some curious anecdotes are related by the possessors as to the manners of these bees; one of which deserves to be recorded. They assert, that at the entrance of each hive a sentinel is placed to watch the outgoings and incomings of his fellows, and that this sentinel is relieved at the expiration of twenty-four hours, when another assumes his post and duties for the same period. Of the duration of this guard some doubts may be reasonably entertained; but of its existence ample evidence has been obtained by repeated observation. At all times a single bee was seen occupying the hole leading to the nest, who, on the approach of another, withdrew himself within a small cavity apparently made for this purpose on the left hand side of the aperture, and thus allowed the passage of the individual entering or quitting the hive, the sentinel constantly resuming his station immediately after the passage had been effected. During how long a time the same individual remained on duty could not be ascertained; for, although many attempts were made to mark him by introducing a pencil tipped with paint, he constantly eluded the aim taken. With the paint thus attempted to be applied to the bee, the margin of the opening was soiled, and the sentinel, as soon as he was free from the annoyance he suffered from the thrusts repeatedly made at its body, approached the foreign substance to taste it, and, evidently disliking the material, he withdrew into his hive. A troop of bees was soon observed to advance towards the place, each individual bearing a small particle of wax, or of propolis, in his mandibles, which he deposited in his turn upon the soiled part of the wood. The little labourers then returned to the hive, and repeated the operation until a small pile rose above the blemished part, and consequently relieved the inhabitants from the annoyance.—*Beechey's Voyage, App.* p. 613, and *Journal of Royal Institution.*

### INTELLIGENCE.

